# Effect of Hydrogel Stiffness on Chemoresistance of Breast Cancer Cells in 3D Culture

**DOI:** 10.3390/gels10030202

**Published:** 2024-03-17

**Authors:** Tianjiao Zeng, Huajian Chen, Toru Yoshitomi, Naoki Kawazoe, Yingnan Yang, Guoping Chen

**Affiliations:** 1 Research Center for Macromolecules and Biomaterials, National Institute for Materials Science, Tsukuba 305-0044, Japan; zeng.tianjiao@nims.go.jp (T.Z.); chen.huajian@nims.go.jp (H.C.); yoshitomi.toru@nims.go.jp (T.Y.); kawazoe.naoki@nims.go.jp (N.K.); 2 Graduate School of Science and Technology, University of Tsukuba, Tsukuba 305-8577, Japan; 3 Graduate School of Life and Environmental Science, University of Tsukuba, Tsukuba 305-8572, Japan; yo.innan.fu@u.tsukuba.ac.jp

**Keywords:** agarose hydrogel, stiffness, chemoresistance, breast cancer cells, 3D culture

## Abstract

Chemotherapy is one of the most common strategies for cancer treatment, whereas drug resistance reduces the efficiency of chemotherapy and leads to treatment failure. The mechanism of emerging chemoresistance is complex and the effect of extracellular matrix (ECM) surrounding cells may contribute to drug resistance. Although it is well known that ECM plays an important role in orchestrating cell functions, it remains exclusive how ECM stiffness affects drug resistance. In this study, we prepared agarose hydrogels of different stiffnesses to investigate the effect of hydrogel stiffness on the chemoresistance of breast cancer cells to doxorubicin (DOX). Agarose hydrogels with a stiffness range of 1.5 kPa to 112.3 kPa were prepared and used to encapsulate breast cancer cells for a three-dimensional culture with different concentrations of DOX. The viability of the cells cultured in the hydrogels was dependent on both DOX concentration and hydrogel stiffness. Cell viability decreased with DOX concentration when the cells were cultured in the same stiffness hydrogels. When DOX concentration was the same, breast cancer cells showed higher viability in high-stiffness hydrogels than they did in low-stiffness hydrogels. Furthermore, the expression of P-glycoprotein mRNA in high-stiffness hydrogels was higher than that in low-stiffness hydrogels. The results suggested that hydrogel stiffness could affect the resistance of breast cancer cells to DOX by regulating the expression of chemoresistance-related genes.

## 1. Introduction

Breast lumps are common in the diagnosis of breast diseases. Around 80% of breast cancer patients seek medical consultations for palpable breast lumps, as the breast cancer tissue is more rigid than their surrounding normal tissue [1,2]. This pathological symptom indicates that an increase in matrix stiffness plays an important role during breast tumorigenesis and the progression of the disease. At the microscopic scale, the stiffness of the matrix, especially the stiffness of the extracellular matrix (ECM), has been found to have a close relationship with cell behavior [3,4].

Matrix stiffness is primarily induced by the rearrangement, cross-linking and deposition along with the degradation of specific ECM proteins. The ECM components in cancers are mainly secreted by cancer-associated fibroblasts (CAFs) [5]. The expression of lysyl oxidase (LOX) by CAFs that initiates the cross-linking of collagen can increase ECM stiffness and further affect cell functions [6,7]. For example, the stiffened ECM drives the activation and stabilization of vinculin and enhances Akt signaling to promote cancer progression [8]. Moreover, ECM stiffness regulates transforming growth factor (TGF)-β-induced epithelial-mesenchymal transition (EMT), fostering cancer cell intravasation and metastasis [9,10]. In light of the crucial role of ECM stiffness in regulating cancer cell functions, extensive research has focused on developing innovative culture platforms with pathologically relevant stiffness in order to enable the in-depth exploration of matrix stiffness-mediated tumorigenesis and cancer progression [11,12,13].

The ECM stiffness in normal breast tissue is less than 1 kPa, whereas it increases to a range of 4 to 100 kPa for breast cancers [14,15,16,17]. Additionally, benign breast masses exhibit much lower stiffness (approximately 39.4 kPa) compared to malignant breast masses (around 100 kPa) [18,19,20]. In a word, during the development of breast cancers, there is a wide-range change in ECM stiffness. The increase in stiffness has been used not only as a diagnostic factor of breast cancers, but also to assess the malignant level [19,20,21,22]. The large variations in stiffness within different pathological grading reveal that cell functions are highly related with ECM stiffness. However, many studies have reported the effects of stiffness on breast cancer cell functions within a narrow range of stiffness that is either 0.2 to 2 kPa [23] or 10 to 57 kPa [24]. There are no comparable results regarding the effect of stiffness across the entire spectrum of breast cancer stiffness in three-dimensional (3D) culture [25]. Given the wide spectrum of ECM stiffness, it is challenging to accurately and comprehensively recapitulate the effect of ECM stiffness on breast cancer development and even the occurrence of chemoresistance of breast cancer cells.

On the other hand, chemotherapy is one of the most common strategies for cancer treatment, whereas drug resistance reduces the chemotherapy efficiency seriously and even causes the failure of chemotherapy [26,27]. The mechanism of chemoresistance emerging is complex, which includes, while not limited to DNA repair, apoptosis and/or autophagy inhibition, expression change in miRNA and overexpression of ATP-binding cassette (ABC) drug transporter proteins [28,29,30,31]. Among them, the enhanced drug efflux out of the cells through the ABC drug transporter protein family, especially overexpression of P-glycoprotein (P-gP, also known as ABCB1), is regarded as the major factor that contributes to drug resistance [32]. Doxorubicin (DOX) is a commonly used first-line drug for breast cancer chemotherapy, while it is also one of the substrates of P-gP [33]. P-gP can trigger DOX efflux out of the cells and therefore induce DOX-resistance in breast cancers [34]. In an early study, chemoresistance was more considered to be the cellular mechanism but with little relationship to the surrounding environment [35]. However, some recent studies have revealed the important role of ECM in the generation of drug resistance [36,37]. For instance, interaction between acute myelogenous leukemia and fibronectin in bone marrow reduces chemosensitivity of the cells [38] and the chemoresistance mediated by receptor–ligand interaction of the cells with ECM is termed as cell-adhesion-mediated drug resistance (CAM-DR) [39]. Although numerous studies have reported the effect of ECM on chemoresistance, the relationship between ECM stiffness and chemoresistance occurrence is unknown. Investigating the effect of ECM stiffness on the chemoresistance of breast cancer cells can help to deepen our understanding of the mechanism of chemoresistance and to improve the treatment of drug-resistant cancers.

Hydrogels are ideal materials to create ECM conditions because they can be fabricated from various artificial or natural polymers and can span the range of stiffness seen in tissues and organs. Agarose hydrogel is one of the most common hydrogels that has been used in biomedical research. Agarose is a linear polysaccharide extracted from seaweed and has good biocompatibility for cell culture and tissue engineering applications [40,41,42]. Unlike the gelation process of other hydrogels, agarose hydrogels can be easily formed through temperature controlling [43,44]. At high temperatures, agarose exists in the form of random coils in aqueous solutions [45]. As the temperature decreases to the gelation point, the agarose helices are associated with long fiber-like aggregates that eventually form a percolating network during the sol–gel transition [46,47]. After gelation, the hydrogels remain stable at 37 °C and there is no need to introduce other cross-linking agents. More importantly, the mechanical properties of agarose hydrogels, such as stiffness, are highly dependent on the concentration and molecular weight [45,46,48]. Thus, the properties of agarose hydrogel can be easily tuned to recapitulate the mechanical characteristics of the ECM in breast cancers. Agarose hydrogels are considered as a suitable platform to study the effect of stiffness on breast cancer cell function in 3D culture.

**Figure 1:**
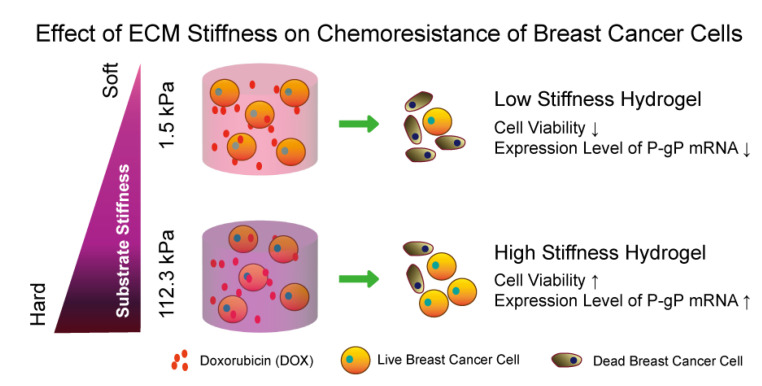
Usage of agarose hydrogels to mimic the ECM stiffness of breast cancers to investigate the effect of ECM stiffness on chemoresistance of breast cancer cells.

Therefore, in this study, agarose hydrogels with a wide range of stiffness (1.5 kPa~112.3 kPa) were prepared to cover the whole stiffness range of breast cancers and used to encapsulate breast cancer cells for 3D culture. The effect of agarose hydrogel stiffness on the resistance of breast cancer cells to DOX was investigated. The possible mechanism was elucidated through analyzing the expression of P-gP mRNA (Figure 1).

## 2. Results and Discussion

### 2.1. Preparation and Characterization of Agarose Hydrogels

**Figure 2:**
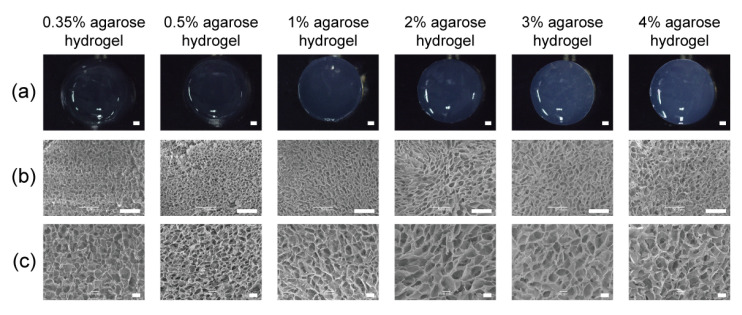
Gross appearance (**a**) and SEM image of agarose hydrogels (**b**,**c**) prepared from 0.35%, 0.5%, 1%, 2%, 3% and 4% agarose aqueous solutions. Scale bar: 1 mm (**a**), 500 μm (**b**) and 100 μm (**c**).

The agarose hydrogels were prepared from 0.35%, 0.5%, 1%, 2%, 3% and 4% agarose aqueous solutions. Their gross appearance is shown in Figure 2a. The hydrogels were transparent with increasing turbidity as the concentration of agarose increased. The SEM observation of the lyophilized agarose hydrogels showed that all the hydrogels had similar porous structures (Figure 2b,c). The porous structures were formed after lyophilization. The SEM images only showed the microporous structures of the lyophilized hydrogels, not the mesh size of the hydrated hydrogels.

**Figure 3:**
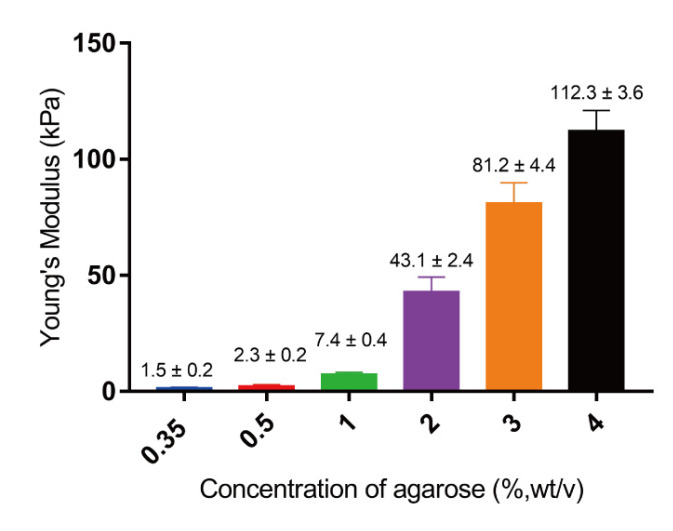
Young’s modulus of agarose hydrogels prepared from different concentrations of agarose. Data are the means ± S.D. (*n* = 5).

The stiffness of agarose hydrogels was measured using a compressive strength analysis. As presented in Figure 3, the stiffness increased with the increase in agarose concentration. The 0.35% agarose hydrogel had the lowest stiffness (1.5 ± 0.2 kPa) and 4% had the highest stiffness (112.3 ± 3.6 kPa). The increase in stiffness should be due to the dense agarose networks in the hydrogels of high concentrations. The stiffness of these agarose hydrogels could cover the ECM stiffness range of breast cancers (4 kPa~100 kPa). The hydrogels were used to encapsulate breast cancer cells for 3D culture to investigate the effect of hydrogel stiffness on their resistance to DOX.

### 2.2. Effect of Agarose Hydrogel Stiffness on Chemoresistance of Breast Cancer Cells to DOX

**Figure 4:**
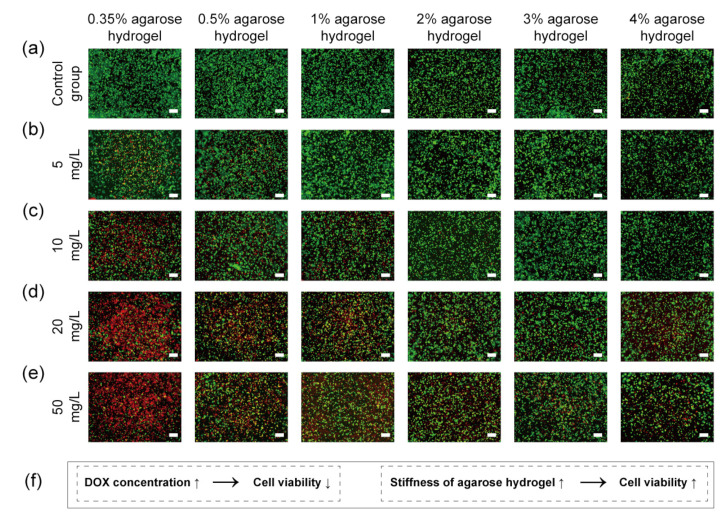
Live/dead staining of breast cancer cells after 24 h culture in 0.35%, 0.5%, 1%, 2%, 3% and 4% agarose hydrogels without (**a**) or with different concentrations of DOX (**b**–**e**). A brief summary illustration of live/dead staining results (**f**). Scale bar: 200 μm. Green fluorescence: live cells; red fluorescence: dead cells.

MDA-MB-231 cells were encapsulated in the agarose hydrogels with or without DOX. The agarose hydrogels with DOX were prepared by mixing DOX in the agarose solutions before gelation. The cells encapsulated in the hydrogels were cultured in medium supplemented with or without DOX for 24 h. Afterward, cell viability was investigated using live/dead staining (Figure 4).

Almost all the breast cancer cells cultured in the agarose hydrogels without DOX were alive (green fluorescence) (Figure 4a). The results indicated that the agarose hydrogels without DOX were nontoxic to cells. When DOX was added, some dead cells (red fluorescence) were observed. The number of dead cells was dependent on both DOX concentration and agarose hydrogel stiffness (Figure 4). When DOX concentration was low (5 mg L^−1^), a small number of dead cells were observed in the 0.35% and 0.5% agarose hydrogels, whereas few dead cells were observed in the agarose hydrogels prepared with concentrations of 1%, 2%, 3% and 4% (Figure 4b). When DOX concentration increased to 10 mg L^−1^, more dead cells were observed in the 0.35% and 0.5% agarose hydrogels and a small number of dead cells were observed in the 1% agarose hydrogel, while few dead cells were observed in the 2%, 3% and 4% agarose hydrogels (Figure 4c). With the further increase in DOX concentration to 20 mg L^−1^ and 50 mg L^−1^, most cells were dead in the 0.35% agarose hydrogels. A large number of dead cells were observed in the 0.5% and 1% agarose hydrogels. Some dead cells were also observed in the 2%, 3% and 4% agarose hydrogels (Figure 4d,e). The live/dead staining results indicated that the number of live cells decreased with DOX concentration, while they increased with the hydrogel stiffness, as summarized in Figure 4f.

**Figure 5:**
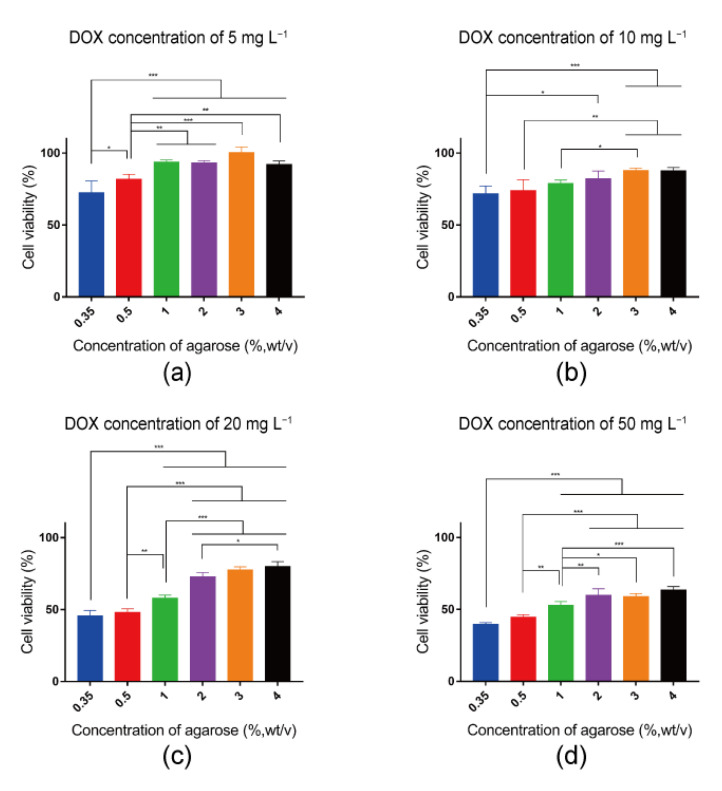
Quantified viability of breast cancer cells after 24 h culture in 0.35%, 0.5%, 1%, 2%, 3% and 4% agarose hydrogels with different DOX concentrations of 5 mg L^−1^ (**a**), 10 mg L^−1^ (**b**), 20 mg L^−1^ (**c**) and 50 mg L^−1^ (**d**). The data were normalized to the cell viability in the respective agarose hydrogels without DOX. Data are the means ± S.D. (*n* = 3). Significant differences: * *p* < 0.1; ** *p* < 0.01; *** *p* < 0.001.

Cell viability was furthered quantified with an AlamarBlue assay (Figure 5). At the same concentration of DOX, cell viability increased with the increase in hydrogel stiffness. When DOX concentration was fixed at 5 mg L^−1^, 70% of cells were alive in the 0.35% agarose hydrogel, which was significantly lower than that observed in other high concentrations of agarose hydrogels (Figure 5a). The cell viability in the 0.5% agarose hydrogel was around 80%, also significantly lower than that in 1%, 2%, 3% and 4% agarose hydrogels. In agarose hydrogels with concentrations of 1%, 2%, 3% and 4%, the cell viability was over 90%. As the concentration of DOX increased to 10 mg L^−1^, the cell viability significantly decreased in all samples (Figure 5b) and exhibited a gradual decrease corresponding to the decreasing concentration of agarose hydrogel. A low concentration of agarose hydrogel was associated with low cell viability, while a high concentration of agarose hydrogel was associated with high cell viability. When the concentration of DOX increased to 20 mg L^−1^, the decrease in cell viability became more evident (Figure 5c). More than 50% of cells were dead in 0.35% and 0.5% agarose hydrogels, while around 43% of cells were dead in 1% agarose hydrogel. As the concentration of DOX increased to 50 mg L^−1^, the cell viability in all agarose hydrogels decreased continuously (Figure 5d). All these results suggested that cells cultured in the high-stiffness agarose hydrogels were less sensitive to DOX. The increase in stiffness led to a decrease in cell sensitivity to DOX, emphasizing the effect of hydrogel stiffness on the cellular response to the anti-cancer drug.

### 2.3. Effect of Agarose Hydrogel Stiffness on Chemoresistance of Breast Cancer Cells after Different Culture Time

**Figure 6:**
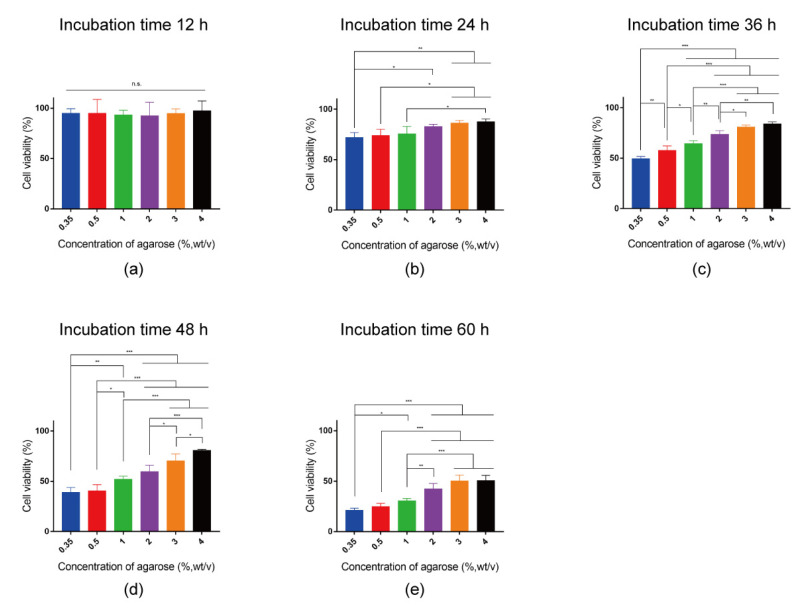
Quantified viability of breast cancer cells after 12 (**a**), 24 (**b**), 36 (**c**), 48 (**d**) and 60 h (**e**) culture in 0.35%, 0.5%, 1%, 2%, 3% and 4% agarose hydrogels containing 10 mg L^−1^ DOX. The data were normalized to the cell viability in the respective agarose hydrogels without DOX. Data are the means ± S.D. (*n* = 3). Significant differences: * *p* < 0.1; ** *p* < 0.01; *** *p* < 0.001. n.s. = no significant difference.

The chemoresistance of breast cancer cells cultured in the agarose hydrogels for different times (12~60 h) was further investigated by fixing the DOX concentration at 10 mg L^−1^ (Figure 6). A concentration of 10 mg L^−1^ DOX was used because this concentration of DOX showed a moderate cytotoxic effect to breast cancer cells cultured in the agarose hydrogels (Figure 4 and Figure 5). In addition to the effect of the stiffness on chemoresistance to different concentrations of DOX, the effect of the stiffness on cellular chemoresistance became more pronounced with prolonged incubation time. After 12 h of culturing at a DOX concentration of 10 mg L^−1^, no significant difference was observed and cell viability in all agarose hydrogels were higher than 90% (Figure 6a). This should be because that the doubling time of MDA-MB-231 cells is around 24 to 25 h [49] and the cytotoxic effect of DOX could not be observed within the 12 h culture. However, after 24 h of culturing, cell viability in all samples decreased and the cell viability had a more pronounced decrease in the low-stiffness hydrogel (Figure 6b). After 36 h of culturing, the cell viability in the 0.35% agarose hydrogel was lower than 50%. The cell viability in the 0.5% agarose hydrogel was significantly higher than that in the 0.35% agarose hydrogel, but significantly lower than that in the other high concentration agarose hydrogels. The cell viability in the 1% agarose hydrogel was around 64%, significantly lower than that in the 2%, 3% and 4% agarose hydrogels. The cell viability in the 2% agarose hydrogel was around 70% and that in the 3% and 4% agarose hydrogels was over 80% (Figure 6c). After 48 h of culturing, the cell viability was lower than 50% in both 0.35% and 0.5% agarose hydrogels, and significantly lower than that in the 1%, 2%, 3% and 4% agarose hydrogels. (Figure 6d). When the culture time was prolonged to 60 h, the cell viability in most hydrogels decreased rapidly, except in the 3% and 4% agarose hydrogels, where approximately 50% of the cells remained alive (Figure 6e).

The results indicated that the cell viability decreased both dose-dependently and incubation time-dependently. Furthermore, the sensitivity of breast cancer cells to DOX was dependent on the stiffness of agarose hydrogels. There were more dead cells in the low-stiffness agarose hydrogels than in the high-stiffness agarose hydrogel. High-stiffness hydrogels could decrease the sensitivity to DOX, while low-stiffness hydrogels could increase the sensitivity to DOX for all dosages of DOX and all culture times. Therefore, it is reasonable to hypothesize that breast cancer cells in stiff ECM could have a higher possibility for emerging chemoresistance ability than the cells in soft ECM.

### 2.4. Effect of Agarose Hydrogel Stiffness on Gene Expression of P-Glycoprotein

**Figure 7:**
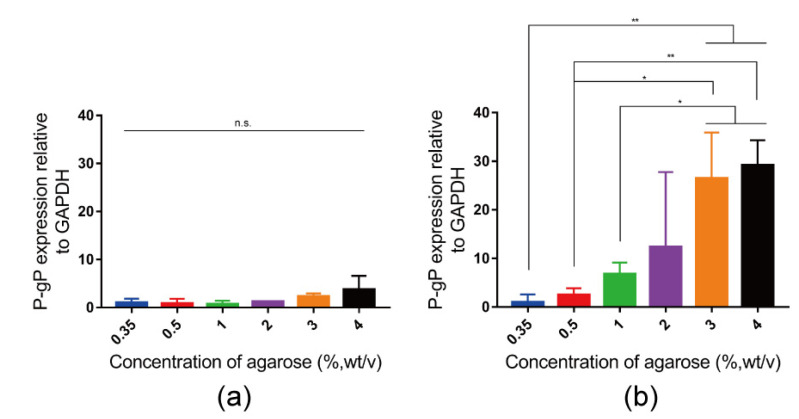
Quantified expression level of P-gP mRNA in breast cancer cells after 36 h culture in 0.35%, 0.5%, 1%, 2%, 3% and 4% agarose hydrogels without DOX (**a**) or with 10 mg L^−1^ DOX (**b**). The data relative to GAPDH were normalized to the expression level in 0.35% agarose hydrogel. Data are the means ± S.D. (*n* = 3). Significant differences: * *p* < 0.1; ** *p* < 0.01. n.s. = no significant difference.

To investigate the possible mechanism of the effect of stiffness on chemoresistance, the expression of P-gP mRNA was analyzed using RT-qPCR. After the breast cancer cells were cultured in hydrogels without or with DOX for 36 h, the expression level of P-gP mRNA was analyzed. For the cells cultured in agarose hydrogels without DOX, the expression level of P-gP mRNA had no significant difference among the agarose hydrogels of different concentrations (Figure 7a). However, when cells were cultured in agarose hydrogels with DOX, the expression level of P-gP mRNA increased compared to the cell cultured in agarose hydrogels without DOX. The expression of P-gP mRNA in 3% and 4% agarose hydrogels was significantly higher than that in 0.35%, 0.5% and 1% agarose hydrogels (Figure 7b). These results demonstrated that stiffness had no effect on the expression of P-gP mRNA without DOX, while stiffness had a positive effect on the expression level of P-gP mRNA at the presence of DOX. The expression of P-gP mRNA in high-stiffness hydrogel increased compared to that in low-stiffness hydrogel. It was worth noting that the expression level of P-gP mRNA increased gradually but showed no significant difference among the 0.35%, 0.5%, 1% and 2% agarose hydrogels. This might be because the effect of stiffness on the expression level of P-gP mRNA had a cumulative effect following the increase in stiffness. The low-stiffness hydrogel lacked a significant effect on the expression of P-gP mRNA. When the stiffness of hydrogels increased, the expression level of P-gP mRNA could increase gradually and become significant.

ECM components play an important role in orchestrating cell functions through cell-matrix cross-talks [50]. In this study, agarose hydrogels of different concentrations were successfully prepared to provide a large range of stiffness (1.5 kPa~112.3 kPa) for 3D cell culture, allowing recapitulation of the wide range of pathological ECM stiffness in breast cancers. Moreover, since the stiffened ECM can act as a physical barrier that limits drug penetration [51], DOX was mixed with the agarose solution before gelation. Therefore, DOX was impregnated in the hydrogels to avoid the diffusion problem. The breast cancer cells were encapsulated in the agarose hydrogels and cultured in the DOX-containing medium in 3D to investigate the effect of stiffness on chemoresistance to anti-cancer drug.

Increase in hydrogel stiffness changed the sensitivity of breast cancer cells to DOX. A stiffened hydrogel was found to decrease the sensitivity of cells to drug, which is consistent with previous reports [23,52,53]. Furthermore, the expression of P-gP mRNA increased when cells were incubated in the agarose hydrogels with DOX, which should be because cancer cells exposing to DOX can stimulate P-gP expression [34]. The interesting result is that the increased level of P-gP mRNA in different stiffness hydrogels was different. In the high-stiffness agarose hydrogel, the P-gP expression level increased more compared to the low-stiffness agarose hydrogel. This upregulation of P-gP mRNA might lead to a high expression of P-gP protein, thereby failing to make DOX accumulate and causing the chemoresistance of breast cancer cells in stiffer hydrogel [32].

## 3. Conclusions

In this study, agarose hydrogels of a wide range of stiffness (1.5 kPa~112.3 kPa) were prepared from different concentrations of agarose aqueous solutions. The hydrogels were used for the 3D culture of breast cancer cells to investigate the effect of hydrogel stiffness on the chemoresistance of breast cancer cells. Cell viability was dependent on hydrogel stiffness, DOX concentration and culture time. Most importantly, the high-stiffness hydrogel containing DOX increased cell viability and promoted expression of P-gP mRNA. The results suggested that ECM stiffness might contribute to the development of chemoresistance of breast cancer cells. Stiffened ECM could induce chemoresistance in breast cancer cells through upregulating the expression of P-gP mRNA.

## 4. Materials and Methods

### 4.1. Preparation and Characterization of Agarose Hydrogels

Different concentrations of agarose solutions ((0.35%, *wt*/*v*), (0.50%, *wt*/*v*), (1.00%, *wt*/*v*), (2.00%, *wt*/*v*), (3.00%, *wt*/*v*) and (4.00%, *wt*/*v*)) were prepared through dissolving autoclave-sterilized agarose powder (A2576, Sigma, St. Louis, MO, USA) in sterilized PBS (1X) under an oil bath at 110 °C for 15 min. Subsequently, the agarose solutions were transferred to a 37 °C water bath to equilibrate the temperature overnight. After temperature equilibration, the agarose solutions were pipetted into a silicon frame having a central hole of φ6 mm × H5 mm. Then, the samples were transferred to a 4 °C refrigerator, enabling gelation for 30 min. After gelation, the silicon frame was removed and the gross appearances of the agarose hydrogels with different concentrations were observed using an optical microscope (Olympus, Tokyo, Japan). Afterward, the agarose hydrogels were transferred to a −80 °C refrigerator for freezing and lyophilized in a freeze-drying machine. The pore structures of the lyophilized agarose hydrogels were examined using scanning electron microscopy (SEM; Hitachi S-4800, Tokyo, Japan).

### 4.2. Stiffness Measurement of Agarose Hydrogels

The stiffness of agarose hydrogels was measured using a static compression test as previously described [54]. Briefly, round discs of agarose hydrogels (φ10 mm × H5 mm) were prepared for the measurement. The preparation of agarose solution was the same as described above. After gelation, a 10 mm diameter biopsy punch was used to cut the hydrogel into discs with 5 mm height. Subsequently, the hydrogel discs were placed at room temperature overnight for temperature equilibration before the mechanical testing. The stress–strain curves of the agarose hydrogel discs were obtained by using a TA.XT. plus Texture Analyzer (Hamilton, MA, USA, test speed = 0.1 mm/s). The Young’s modulus was determined by fitting a line to the linear region of the stress–strain curve. Five samples were used for each measurement.

### 4.3. Cell Culture

The Triple Negative Breast Cancer Cell line MDA-MB-231-Luc (JCRB, Osaka, Japan) were cultured in DMEM serum medium containing 10% fetal bovine serum (FBS, Gibco, Norristown, PA, USA), L-glutamine (Sigma) and antibiotics (100 U mL^−1^ penicillin and 100 μg mL^−1^ streptomycin) in a humidified incubator (5% CO_2_, 37 °C).

### 4.4. In Vitro Anticancer Effect of Doxorubicin in Agarose Hydrogels

The effect of hydrogel stiffness on the anticancer effect of DOX was investigated through culturing the breast cancer cells in agarose hydrogels of different concentrations. All samples were prepared on a clean bench to avoid contamination. DOX at a concentration of 1 g L^−1^ was prepared through dissolving DOX powder into sterilized water and diluting to the required concentration when used. Different concentrations of agarose solutions ((0.41%, *wt*/*v*), (0.58%, *wt*/*v*), (1.17%, *wt*/*v*), (2.33%, *wt*/*v*), (3.50%, *wt*/*v*), (4.67%, *wt*/*v*)) were prepared as described above. After temperature equilibration, 90 μL of agarose solution was mixed with a pre-warmed 10 μL DOX solution to prepare agarose hydrogels at a final DOX concentration of 5, 10, 20 and 50 mg L^−1^. As for the control group without DOX, PBS (1×) with the same volume was used as a replacement for DOX.

Silicon discs with a dimension of φ35 mm × H1 mm and silicon frames having a central hole of φ6 mm × H2 mm were sterilized via autoclaving at 120 °C for 20 min. Nylon mesh (250 μ, Iwai Chemicals Company, Tokyo, Japan) was used to support the agarose hydrogels. The Nylon mesh was cut into round discs (φ6 mm), sterilized with 70% ethanol and washed with PBS (1×) three times. After removing the PBS from the Nylon mesh disc, it was placed on the silicon disc. And then, the silicon frame (φ6 mm × H2 mm) was placed on the Nylon mesh disc. The subcultured MDA-MB-231-Luc cells were detached from the culture plate using trypsin-EDTA solution and re-suspended in DMEM serum medium at a concentration of 1 × 10^7^ cells mL^−1^. Afterward, a 5 μL cell suspension solution was added into the agarose/DOX solution and mixed well. The cell/agarose/DOX mixture solution was pipetted into the silicon frame. The construct was then placed in a 4 °C refrigerator rapidly, enabling gelation for 30 min. After that, the silicon frame was removed, and the cells/agarose/DOX construct together with the Nylon mesh disc were transferred to a 24-well cell culture plate and cultured for 24 h. For the control group, the procedures were the same as the above mentioned, aside from changing DOX to an equal volume of PBS.

### 4.5. Cell Viability Assays

The cell viability in agarose hydrogel was analyzed by using live/dead staining assay and AlamarBlue cell viability quantification. For live/dead staining, the cells were labeled with a live/dead kit (Dojindo, Kumamoto, Japan). Following the staining of the samples with calcein-AM and propidium iodide in serum-free DMEM medium for 10 min at 37 °C, the hydrogels were washed 3 times with PBS and immediately analyzed by using fluorescence microscopy (Olympus, Japan).

AlamarBlue (Thermo Fisher Scientific, Inc., Tokyo, Japan) was used to assess the metabolic activity of cells in 3D culture. The reduction in AlamarBlue resazurin to pink colored resorufin was determined through fluorescence measurement (λex: 530 nm, λem: 590 nm). All measurements were conducted in a microplate reader (Spark Multimode Microplate Reader, Tecan) using a 96-well black-clear flat bottom plate (Nunclon Delta-Treated, Flat-Bottom Microplate, Thermo Fisher Scientific, Inc., Japan). For the details, after the cell culturing at different times, the cells/agarose and cells/agarose/DOX constructs were transferred to a new 24-well plate and 1 mL AlamarBlue working solution (100 μL of AlamarBlue diluted with 900 μL fresh DMEM medium) was added to each well, followed by incubation at 37 °C for 4 h. Subsequently, 100 μL of the working solution was transferred to the 96-well black-clear flat bottom plate to measure the fluorescence intensity. The value of the fluorescence intensity was used to calculate the cell viability. The cell viability of the cells/agarose/DOX hydrogels was normalized to the cell viability in the respective agarose hydrogel without DOX. Three samples were used for each measurement to calculate the means and standard deviations.

### 4.6. Real-Time PCR Analysis (RT-qPCR Analysis)

The P-glycoprotein mRNA expression levels in the cells/agarose and cells/agarose/DOX constructs were analyzed after cell culturing for 36 h using real-time PCR according to a previous method [55,56]. The samples were immersed in 1 mL of a Sepasol solution (Nacalai Tesque, Kyoto, Japan) for extraction of total RNA. A high-capacity cDNA reverse transcription kit (Applied Biosystems, Foster City, CA, USA) was used to reverse transcription of the complementary DNA (cDNA) from 1 μg of purified total RNA. The cDNA served as a template for RT-qPCR analysis and the amplification of glyceraldehyde-3-phosphate dehydrogenase (GAPDH) and P-glycoprotein (ABCB1) was conducted using a QuantStudios 3 Real-Time PCR system (Thermo Fisher Scientific). The pre-designed primer and probe sequences of ABCB1 (Hs00184500_m1) were used and GAPDH (Hs99999905_m1) was used as the housekeeping gene (Thermo Fisher, Japan). The relative expression of each gene was calculated using a 2^−∆∆Ct^ method with an endogenous control (GAPDH). The expression level of ABCB1 in each group was normalized using the respective gene expression of the cells cultured in a 0.35% concentration of agarose hydrogel as a reference. Three samples were used for each measurement to calculate the means and standard deviations.

### 4.7. Statistical Analysis

All quantitative experiments were repeated in triplicate or quintuplicate to calculate the means and standard deviations (S.D.). Statistical analysis of the experimental data was performed using GraphPad Prism software. One-way ANOVA with Dunnett’s multiple comparison test were used to compare the samples. The p value was used to determine the level of significance: * *p* < 0.05, ** *p* < 0.01, and *** *p* < 0.001.

## Data Availability

All data and materials are available on request from the corresponding author. The data are not publicly available due to ongoing researches using a part of the data.
